# Multivariate predictive model for predicting in-hospital mortality in HIV-associated talaromycosis: a multicenter retrospective study

**DOI:** 10.1371/journal.pntd.0014432

**Published:** 2026-06-08

**Authors:** Zhikai Wan, Mengyan Wang, Weiwei Zhang, Yu Zhou, Biao Zhu

**Affiliations:** 1 The Department of Infectious Diseases, State Key Laboratory for Diagnosis and Treatment of Infectious Diseases, National Clinical Research Center for Infectious Diseases, China-Singapore Belt and Road joint Laboratory on Infection Research and Drug Development, National Medical Center for Infectious Diseases, Collaborative Innovation Center for Diagnosis and Treatment of Infectious Diseases, The First Affiliated Hospital, School of Medicine, Zhejiang University, Hangzhou, China; 2 Xixi Hospital of Hangzhou, Hangzhou, China; 3 Department of Infectious Disease, The Third Affiliated Hospital of Wenzhou Medical University, Wenzhou, China; Gulu University, UGANDA

## Abstract

**Background:**

Talaromycosis is an invasive fungal infection that predominantly affects immunocompromised individuals, with a particularly high incidence and mortality rate among HIV-infected patients. The purpose of this study was to develop and validate a novel nomogram model to predict mortality risk in HIV-associated talaromycosis (HTM) patients.

**Method:**

The authors retrospectively analyzed HTM patients from January 2013 to December 2023 at three research centers. The research participants were randomly divided into the training and validation sets at a ratio of 7:3. To determine the crucial variables for establishment of the predictive model, the study sequentially applied univariate logistic regression, lasso regression, stepwise logistic regression. The validation set was used to assess the performance of the established prediction model, with its efficacy evaluated through receiver operating characteristics curve, clinical decision curves, and calibration curves.

**Result:**

A total of 431 subjects were enrolled in the study with 55/431 (12.76%) patients dying during hospitalization. Statistical analysis shows that there was no difference between training set and validation set in the baseline demographic and clinical characteristics. Five factors including breathlessness, elevated TB, APRI, CRP and decreased Hb were identified as predictive factors for HTM mortality. A nomogram model was built and the area under the curve (AUC) for the nomogram in predicting death was 0.83 (95% CI: 0.76-0.90) in the training set and 0.81 (95% CI: 0.70-0.93) in the validation set. The H-L test and calibration curves showed a strong alignment between predicted and actual results in both sets. Additionally, the decision curve analysis (DCA) indicated that the model provided significant net benefits for patients experiencing poor outcomes.

**Conclusion:**

The nomogram model developed in this study integrating easily accessible clinical indicators and symptoms is effective in predicting in-hospital mortality in patients with HTM, which will greatly assist clinicians in the individual management of HTM patients.

## Author summary

Human immunodeficiency virus (HIV)-associated talaromycosis is a life-threatening invasive fungal infection that remains a major cause of mortality among immunocompromised individuals, yet tools for identifying those at highest risk remain limited. In this multicenter retrospective study of 431 hospitalized patients, we sought to address this gap by developing a practical predictive tool. Using routine clinical and laboratory parameters readily available at the bedside—namely, the presence of breathlessness, elevated total bilirubin, elevated aspartate aminotransferase-to-platelet ratio index (APRI) score, elevated C-reactive protein, and decreased hemoglobin—we constructed a nomogram model to estimate the risk of in-hospital death. The model exhibited good discrimination in both the training and validation cohorts. Given that all included variables are routinely accessible at the time of admission, this nomogram may support clinicians in early risk stratification and individualized patient management.

## Introduction

Talaromycosis, an endemic invasive mycosis, is caused by the pathogenic fungus Talaromyces marneffei primarily prevalent in tropical and subtropical regions of Southeast Asia, especially in Vietnam, Thailand, Northeastern India, and Southern China [1–4]. Talaromyces marneffei is commonly found in immunocompromised individuals, such as those infected with HIV, and is transmitted through spores that enter the body via the respiratory tract or direct contact. As of mid-2022, more than 288,000 cases of talaromycosis have been reported in 34 countries, with an overall prevalence of 3.6% among people living with HIV [5]. In China, the prevalence of talaromycosis among people with HIV (PWH) ranges from 0.2% to 26.5% [6]. It is estimated that by 2050, there will be 4,951 new cases of HIV-associated talaromycosis (HTM) annually in southern regions, with the endemic areas continuing to expand [7].

Talaromycosis can involve multiple organs and systems, with clinical manifestations including fever, anemia, bone marrow involvement, lymphadenopathy, skin lesions, hepatosplenomegaly, respiratory symptoms, and weight loss [8]. Previous studies have indicated that, if left untreated, the mortality associated with talaromycosis in PWH may approach 100% [5]. Despite the availability of effective antifungal therapies, the mortality rate among patients with HTM remains approximately 25% [8]. A study comprising 6,791 individuals infected with HIV demonstrated that patients with Talaromyces marneffei infection experience the highest mortality rate among all acquired immunodeficiency syndrome (AIDS)-related complications (25.0 per 100 person-months, 95% CI 21.5-26.7), representing a statistically significant increase compared to HIV/AIDS patients without Talaromyces marneffei infection (13.8 per 100 person-months, 95% CI 12.5-15.1) [9].

Previous prognosis assessments for HTM rely predominantly on isolated biomarkers such as CD4 + counts, fungal burden measurements [10,11]. Recent studies have demonstrated that decreased albumin and elevated total bilirubin were independent risk factors for poor prognosis of HTM patients [12]. In current clinical practice, there is a lack of individualized prognostic assessment tools for patients with HTM, hindering the early identification of high-risk individuals and limiting the precise allocation of treatment resources. To address this knowledge gap, we conducted a multicenter cohort study to perform a comprehensive analysis of demographic data, coinfections, and laboratory results of HTM patients. The purpose of the study was to identify prognostic risk factors for HTM and develop a clinical prediction model, providing a straightforward method for predicting patient outcomes.

## Method

### Ethics statement

This study was performed in line with the principles of the Declaration of Helsinki. The trial protocol was granted by the Ethics Committees of The First Affiliated Hospital, Zhejiang University School of Medicine, Hangzhou Xixi Hospital and the Third Affiliated Hospital of Wenzhou Medical University (Approval Number: ITT20250096B, HX2024045, YJ2025046). All data were analyzed anonymously. The committee waived the requirement for written informed consent from participants.

### Study design and participants

This was a retrospective and multicenter study of HIV-associated talaromycosis (HTM). From January 1, 2013 to December 1, 2023, cases of HTM admitted to the First Affiliated Hospital, School of Medicine, Zhejiang University, Hangzhou Xixi Hospital and the Third Affiliated Hospital of Wenzhou Medical University were sorted and analyzed. Subjects with the following criteria were included: (i) Age ≥ 18 years; (ii) HIV infection was confirmed by Western blot; (iii) TM infection was confirmed by culture, histopathology, or molecular methods (including Polymerase Chain Reaction or metagenomic Next‑Generation Sequencing) from specimens such as blood, bone marrow, bronchoalveolar lavage fluid (BAL), and/or other body fluid samples. Patients lacking prognostic and comorbidity information or necessary laboratory record were excluded. The enrolled patients were randomly split into a development cohort and a validation cohort at a ratio of 7:3. The development cohort was utilized to establish the predictive model, while the validation cohort was employed to assess the model’s discriminative performance and clinical utility.

### Data collection and pre-processing

A standardized data collection form was designed to extract data from the hospital electronic medical record system including admission and discharge date, demographics (sex and age), symptoms, ART status, co-infection pathogens, laboratory findings (routine blood test, liver function tests, and kidney function tests, hemagglutination index, C-reactive protein (CRP), fungal G test were conducted at admission). Serum creatinine (CR) was dichotomized into normal and elevated groups using sex‑specific upper limits of normal (110 μmol/L for males and 90 μmol/L for females) according to BMJ Best Practice reference ranges [13]. Treatment outcome was also collected. Antifungal treatment data were collected from the electronic medical records. Based on the induction regimen, patients were categorized into three groups: (i) intravenous amphotericin B deoxycholate (AmBd) at 0.7–1.0 mg/kg/day; (ii) intravenous voriconazole (6 mg/kg every 12 hours for the first 24 hours, followed by 4 mg/kg daily); and (iii) oral itraconazole (200 mg every 12 hours) when the other two agents were unavailable. The primary outcome was all‑cause in‑hospital mortality, defined as death from any cause during the index hospitalization. We did not adjudicate the specific cause of death. The exposure factors were the demographic factors and the first laboratory finding upon admission. The participants were stratified into two groups based on hospitalization outcomes: the survival group (patients who achieved clinical stability and survived to discharge) and the death group (those who experienced in-hospital mortality). For continuous variables exhibiting a missing data rate below 5%, missing values were imputed using either the mean or median during data preprocessing. Variables demonstrating a missing proportion exceeding 20% were excluded a priori from the analytical dataset. Variables with intermediate missing rates (5–20%) were subjected to multiple imputation techniques.

### Statistical analyses

Continuous variables were described using the mean (standard deviations (SD)) or median (interquartile range (IQR)), as appropriate. Categorical data were expressed as counts and percentages. The chi-square test, Fisher’s exact test, two-sample t-test (parametric test), or Mann-Whitney U-test (nonparametric test) were used to assess differences between groups in terms of demographic and clinical characteristics. All enrolled patients were randomly assigned into training and validation sets in a 7:3 ratio. To determine potential predictive factors, the training set was subjected to univariate logistic regression and the least absolute shrinkage and selection operator (LASSO) regression method which effectively eliminated irrelevant or highly collinear independent variables. Then a stepwise logistic regression analysis was used to build a prediction model by incorporating the features selected in the LASSO regression model. Variables with a P value greater than 0.05 were excluded from the model during the selection process. The validation set was used to assess the performance of the established prediction model, with its efficacy evaluated through receiver operating characteristic (ROC) curve, clinical decision curves, and calibration curves. Data processing and analysis were performed using R version 4.4.0, along with Zstats 1.0 (www.zstats.net). Two-tailed p-values less than 0.05 were considered to indicate significance.

## Result

### The demographic and clinical characteristics of participants

**Fig 1:**
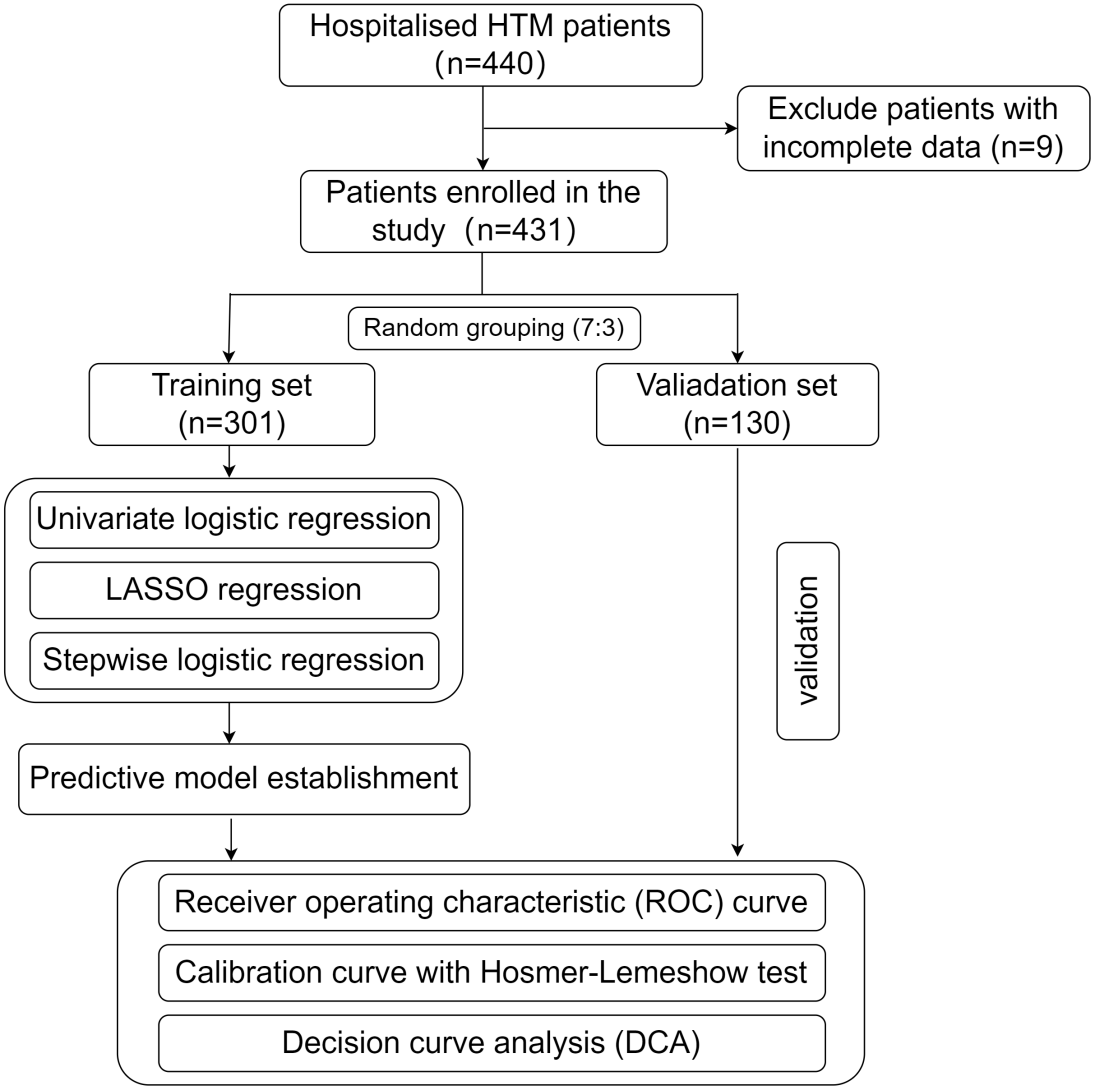
The research flow chart.

A total of 431 patients were enrolled in this study: 301 subjects were assigned to the training set and 130 subjects to the validation set. A flowchart depicting the study population selection and study process is presented in Fig 1. The median age of the enrolled subjects was 38 (IQR 29-51) years and 117/431 (27.15%) were older than 50 years. Among the patients, 89.10% were male. The median CD4 + T cell count was 13 cells/μL (IQR 4.00-38.01). Of these, 341 (79.12%) patients had positive blood cultures, 35 (8.12%) had positive bone marrow cultures, 68 (15.78%) had positive cultures from BALF and 28 (6.50%) had positive histopathology results. The most common symptoms were fever (89.77%), cough (48.84%), rash (20.28%), abdominal pain (16.78%) and breathlessness (14.39%). Regarding induction antifungal therapy, 135 patients (31.32%) received intravenous amphotericin B deoxycholate, 200 (46.40%) received intravenous voriconazole, and 96 (22.27%) received oral itraconazole. Additionally, of all the participants, 55 patients died during hospitalization, with an all-cause mortality rate of 12.76%. Statistical analysis revealed that all baseline characteristics were comparable between the training set and validation set, with no significant differences observed (Table 1).

**Table 1:** Characteristics of the training set and validation set.

Parametric	Total (n = 431)	Training set (n = 301)	Validation set (n = 130)	P
**Age**	38.00 (29.00, 51.00)	38.00 (30.00, 50.00)	40.50 (29.00, 54.00)	0.470
**Age stratification**				0.266
<50	314 (72.85)	224 (74.42)	90 (69.23)	
≥50	117 (27.15)	77 (25.58)	40 (30.77)	
**Gender**				0.539
male	384 (89.10)	270 (89.70)	114 (87.69)	
female	47 (10.90)	31 (10.30)	16 (12.31)	
**Dead**				0.897
No	376 (87.24)	263 (87.38)	113 (86.92)	
Yes	55 (12.76)	38 (12.62)	17 (13.08)	
**TM positive specimens**				
Blood	341 (79.12)	243 (80.73)	98 (75.38)	0.210
Bone Marrow	35 (8.12)	27 (8.97)	8 (6.15)	0.326
BALF	68 (15.78)	47 (15.61)	21 (16.15)	0.888
Tissue	28 (6.50)	19 (6.31)	9 (6.92)	0.813
**G-test**				0.874
positive	218 (50.58)	153 (50.83)	65 (50.00)	
negative	213 (49.42)	148 (49.17)	65 (50.00)	
**ART naïve**				0.225
Yes	328 (76.10)	234 (77.74)	94 (72.31)	
no	103 (23.90)	67 (22.26)	36 (27.69)	
**Induction therapy**				0.457
Amphotericin B deoxycholate	135 (31.32)	92 (30.56)	43 (33.08)	
Voriconazole	200 (46.40)	137 (45.51)	63 (48.46)	
Itraconazole	96 (22.27)	72 (23.92)	24 (18.46)	
**Coinfection**				
PCP	21 (4.87)	13 (4.32)	8 (6.15)	0.417
MTB	38 (8.82)	26 (8.64)	12 (9.23)	0.842
Cryptococcus	9 (2.09)	7 (2.33)	2 (1.54)	0.875
CMV	100 (23.20)	66 (21.93)	34 (26.15)	0.340
EBV	119 (27.61)	78 (25.91)	41 (31.54)	0.231
**Clinical symptom**				
Fever	386 (89.56)	265 (88.04)	121 (93.08)	0.071
Rash	87 (20.19)	62 (20.60)	25 (19.23)	0.761
Breathlessness	62 (14.39)	50 (16.61)	12 (9.23)	0.052
Cough	210 (48.72)	147 (48.84)	63 (48.46)	0.999
Abdominal pain	72 (16.71)	52 (17.28)	20 (15.38)	0.676
**Laboratory test**				
WBC (10^9^/L)	3.83 (2.50, 5.53)	3.66 (2.50, 5.42)	4.13 (2.50, 5.58)	0.688
Hb (g/L)	101.00 (84.00, 115.00)	100.00 (83.00, 115.00)	102.00 (88.50, 115.75)	0.426
PLT (109/L)	115.00 (60.50, 190.50)	120.00 (62.00, 194.00)	101.50 (58.50, 173.00)	0.242
ALB (g/L)	28.60 (23.90, 32.95)	28.70 (23.70, 33.00)	28.55 (24.60, 32.53)	0.963
ALT (U/L)	35.00 (20.00, 65.00)	35.00 (19.00, 65.00)	33.50 (22.25, 64.75)	0.450
AST (U/L)	71.00 (34.50, 131.00)	71.00 (34.00, 136.00)	69.00 (39.08, 122.77)	0.936
AKP (U/L)	105.00 (72.00, 198.50)	105.00 (72.00, 200.00)	105.75 (73.50, 194.75)	0.852
CHE (U/L)	3373.00 (1984.00, 5074.50)	3487.00 (1850.00, 5227.00)	3182.00 (2317.00, 4816.25)	0.808
TB (μmol/L)	9.30 (6.50, 15.00)	9.33 (6.45, 15.00)	9.25 (6.60, 15.00)	0.934
γ-GT (U/L)	79.00 (42.00, 146.50)	76.00 (42.00, 146.00)	83.50 (44.00, 149.75)	0.563
Elevated creatinine	29 (6.73)	23 (7.64)	6 (4.62)	0.347
TG (mmol/L)	1.57 (1.17, 1.93)	1.60 (1.21, 1.96)	1.47 (1.14, 1.80)	0.190
TC (mmol/L)	2.88 (2.21, 3.40)	2.88 (2.24, 3.53)	2.91 (2.21, 3.27)	0.466
LDL (mmol/L)	1.60 (1.02, 2.01)	1.60 (1.03, 2.09)	1.56 (1.00, 1.89)	0.567
HDL (mmol/L)	0.51 (0.32, 0.64)	0.51 (0.32, 0.65)	0.51 (0.34, 0.62)	0.887
LDH (U/L)	399.00 (258.50, 605.50)	391.00 (255.00, 594.00)	435.00 (269.75, 637.25)	0.518
CRP (mg/L)	60.00 (27.61, 97.20)	63.00 (29.26, 100.20)	51.30 (23.88, 84.42)	0.217
PT (s)	13.00 (12.20, 14.00)	13.00 (12.20, 14.10)	12.80 (12.00, 13.80)	0.340
Fibrinogen (g/L)	3.40 (2.47, 4.34)	3.40 (2.47, 4.39)	3.42 (2.50, 4.24)	0.675
D-dimer (μg/L FEU)	6.29 (1.97, 19.12)	5.89 (2.02, 20.00)	7.72 (1.93, 17.88)	0.609
CD3 + cell (cells/μL)	250.00 (143.00, 372.50)	248.00 (148.00, 353.00)	264.50 (129.25, 416.50)	0.997
CD4 + T cell (cells/μL)	13.00 (4.00, 38.01)	12.00 (4.00, 38.01)	13.00 (5.00, 38.01)	0.455
CD8 + T cell (cells/μL)	212.00 (123.50, 299.00)	204.00 (126.00, 290.00)	223.00 (116.25, 310.75)	0.867
CD4/CD8 ratio	0.07 (0.03, 0.15)	0.07 (0.03, 0.15)	0.08 (0.04, 0.15)	0.438

Data are presented as median (interquartile range) or n (%).

Annotation: AKP, Alkaline phosphatase; ALB, Albumin; ALT, Alanine aminotransferase; ART naïve, Antiretroviral therapy naïve; AST, Aspartate aminotransferase; BALF, Bronchoalveolar lavage fluid; CHE, Cholinesterase; CMV, Cytomegalovirus; CR, Creatinine; CRP, C-reactive protein; EBV, Epstein-Barr virus; γ-GT: γ-glutamyl transferase; Hb, Hemoglobin; HDL, High-density lipoprotein; LDH, Lactate dehydrogenase; LDL, Low-density lipoprotein; MTB, Mycobacterium tuberculosis; PCP, Pneumocystis pneumonia; PLT, Platelet count; PT, Prothrombin time; TB, Total bilirubin; TC, Total cholesterol; TG, Triglycerides; WBC, White blood cell.

### Baseline characteristics of the training set population

In the training set, a detailed comparison of clinical features and laboratory variables between the survivor and death groups is presented in Table 2. The median age of the death group was 6 years older than the surviving group (P = 0.037). Additionally, a higher frequency of breathlessness was observed in the deceased group (36.84% vs 13.69%, P < 0.001). No significant associations with prognosis were observed for white blood cell count (WBC), aspartate aminotransferase (ALT), creatinine (Cr), CD4 + T cell count, or CD8 + T cell count in either group. Non-survivors demonstrated significantly lower hemoglobin (Hb) levels and platelet counts (PLT), along with reduced hepatic function (as evidenced by aspartate aminotransferase (AST), cholinesterase (CHE) and total bilirubin (TB) levels), impaired coagulation profiles (prothrombin time (PT) and D-dimer), and elevated inflammatory markers including lactate dehydrogenase (LDH) and C-reactive protein (CRP) when compared with survivors (all P < 0.05).

**Table 2:** Comparison of the demographic and clinical characteristics between survival group and death group in the training cohort.

Parametric	Total (n = 301)	Survivor (n = 263)	Death (n = 38)	P
Age	38.00 (30.00, 50.00)	36.00 (29.00, 49.50)	42.00 (37.25, 51.00)	0.037
Age older than 50 years	77 (25.58)	66 (25.10)	11 (28.95)	0.611
Male				
**TM positivity specimen**				
Blood	243 (80.73)	210 (79.85)	33 (86.84)	0.307
Bone Marrow	27 (8.97)	27 (10.27)	0 (0.00)	0.077
BALF	47 (15.61)	42 (15.97)	5 (13.16)	0.655
Tissue	19 (6.31)	18 (6.84)	1 (2.63)	0.521
Positive G test	153 (50.83)	134 (50.95)	19 (50.00)	0.913
ART naïve	234 (77.74)	203 (77.19)	31 (81.58)	0.543
**Coinfection**				
PCP	13 (4.32)	9 (3.42)	4 (10.53)	0.113
MTB	26 (8.64)	22 (8.37)	4 (10.53)	0.893
Cryptococcus	7 (2.33)	6 (2.28)	1 (2.63)	0.999
CMV	66 (21.93)	55 (20.91)	11 (28.95)	0.263
EBV	78 (25.91)	64 (24.33)	14 (36.84)	0.100
**Clinical symptom**				
Fever	265 (88.04)	234 (88.97)	31 (81.58)	0.296
Rash	62 (20.67)	53 (20.23)	9 (23.68)	0.623
Breathlessness	50 (16.61)	36 (13.69)	14 (36.84)	0.001
Cough	147 (48.84)	127 (48.29)	20 (52.63)	0.617
Abdominal pain	52 (17.28)	44 (16.73)	8 (21.05)	0.510
**Induction therapy**				0.821
Amphotericin B deoxycholate	92 (30.56)	82 (31.18)	10 (26.32)	
Voriconazole	137 (45.51)	119 (45.25)	18 (47.37)	
Itraconazole	72 (23.92)	62 (23.57)	10 (26.32)	
**laboratory test**				
WBC (10^9^/L)	3.66 (2.50, 5.42)	3.66 (2.50, 5.30)	3.70 (2.60, 7.27)	0.427
Hb (g/L)	100.00 (83.00, 115.00)	101.00 (83.00, 116.00)	93.00 (71.25, 105.75)	0.013
PLT (109/L)	120.00 (62.00, 194.00)	127.00 (73.50, 202.00)	75.50 (25.50, 114.75)	0.001
ALB (g/L)	28.70 (23.70, 33.00)	29.30 (24.20, 33.40)	23.70 (21.07, 28.35)	0.001
ALT (U/L)	35.00 (19.00, 65.00)	34.00 (19.00, 63.50)	43.00 (23.00, 70.25)	0.276
AST (U/L)	71.00 (34.00, 136.00)	63.00 (30.50, 123.50)	136.00 (65.75, 234.50)	0.001
AKP (U/L)	105.00 (72.00, 200.00)	98.00 (70.50, 168.95)	200.50 (107.75, 247.00)	0.001
CHE (U/L)	3487.00 (1850.00, 5227.00)	3762.86 (2084.50, 5319.50)	2167.50 (1314.25, 3265.50)	0.001
TB (μmol/L)	9.33 (6.45, 15.00)	8.90 (6.35, 13.15)	16.80 (10.77, 30.57)	0.001
γ-GT (U/L)	76.00 (42.00, 146.00)	68.00 (42.00, 143.50)	111.00 (82.75, 145.75)	0.04
Elevated creatinine	23 (7.64)	14 (5.32)	9 (23.68)	0.001
TG (mmol/L)	1.60 (1.21, 1.96)	1.57 (1.19, 1.90)	1.75 (1.45, 2.22)	0.033
TC (mmol/L)	2.88 (2.24, 3.53)	2.91 (2.29, 3.53)	2.57 (1.71, 3.30)	0.043
LDL (mmol/L)	1.60 (1.03, 2.09)	1.60 (1.12, 2.11)	1.30 (0.65, 1.67)	0.007
HDL (mmol/L)	0.51 (0.32, 0.65)	0.51 (0.35, 0.65)	0.34 (0.16, 0.55)	0.005
LDH (U/L)	391.00 (255.00, 594.00)	377.00 (246.50, 594.00)	594.00 (339.00, 929.25)	0.001
CRP (mg/L)	63.00 (29.26, 100.20)	60.00 (27.26, 91.62)	85.09 (50.55, 152.73)	0.004
PT (s)	13.00 (12.20, 14.10)	12.90 (12.10, 13.90)	13.90 (12.93, 16.88)	0.001
Fibrinogen (g/L)	3.40 (2.47, 4.39)	3.45 (2.65, 4.50)	2.49 (1.58, 3.45)	0.001
D-dimer (μg/L FEU)	5.89 (2.02, 20.00)	5.25 (1.83, 15.16)	17.35 (3.67, 26.53)	0.003
CD3 + cell (cells/μL)	248.00 (148.00, 353.00)	249.00 (150.50, 368.00)	210.00 (114.50, 329.91)	0.308
CD4 + T cell (cells/μL)	12.00 (4.00, 38.01)	12.00 (4.00, 36.00)	10.00 (5.25, 38.01)	0.599
CD8 + T cell (cells/μL)	204.00 (126.00, 290.00)	208.00 (128.00, 306.00)	178.50 (96.75, 271.10)	0.225
CD4/CD8 ratio	0.07 (0.03, 0.15)	0.06 (0.02, 0.15)	0.10 (0.05, 0.15)	0.164
AFR	8.25 (6.73, 10.66)	8.23 (6.75, 10.38)	8.53 (6.41, 16.20)	0.121
APRI	1.50 (0.51, 4.87)	1.29 (0.47, 4.36)	5.49 (1.70, 18.60)	<.001

Data are presented as median (interquartile range) or n (%).

Annotation: AFR, albumin-to-fibrinogen ratio; AKP, Alkaline phosphatase; ALB, Albumin; ALT, Alanine aminotransferase; APRI, aminotransferase/platelet ratio index; ART naïve, Antiretroviral therapy naïve; AST, Aspartate aminotransferase; BALF, Bronchoalveolar lavage fluid; CHE, Cholinesterase; CMV, Cytomegalovirus; CR, Creatinine; CRP, C-reactive protein; EBV, Epstein-Barr virus; γ-GT: γ-glutamyl transferase; Hb, Hemoglobin; HDL, High-density lipoprotein; LDH, Lactate dehydrogenase; LDL, Low-density lipoprotein; MTB, Mycobacterium tuberculosis; PCP, Pneumocystis pneumonia; PLT, Platelet count; PT, Prothrombin time; TB, Total bilirubin; TC, Total cholesterol; TG, Triglycerides; WBC, White blood cell count.

### Risk factors for poor prognosis of HTM

In a univariate logistic regression analysis comparing the survivor group with the non-survivor group it was revealed that breathlessness, WBC, Hb, PLT, ALB, ALT, AST, AKP, CHE, TB, Elevated creatinine, TG, LDL, HDL, LDH, CRP, PT, Fibrinogen, albumin-to-fibrinogen ratio (AFR) and AST-to-platelet ratio index (APRI) were probable valuable prognostic factors in HIV/TM patients. Given the large number of variables involved, a two-step approach was further used to screen for clinical characteristics. Firstly, all potential risk factors with a P value < 0.05 in the univariate logistic analysis were entered into LASSO regression analysis. However, since AFR and APRI (AST-to-platelet ratio index) are composite variables (AFR combines albumin and fibrinogen; APRI combines AST and PLT), they were prioritized in LASSO regression to mitigate covariance and accommodate the limited sample size. As shown in S1 Fig, 16 potential risk factors were included in LASSO and nine candidate predictors were determined including breathlessness, WBC, *Hb*, TB, Elevated creatinine, TG, CRP, PT, APRI. Subsequently, multivariate logistic regression was performed on these nine feature predictors. The result indicated that symptom of breathlessness (OR=5.80, 95% CI: 2.46 ~ 13.69), baseline Hb (OR=0.98, 95%CI: 0.96 ~ 0.99), TB (OR=1.02, 95%CI:1.01 ~ 1.03), CRP (OR=1.01, 95%CI:1.01 ~ 1.01), APRI (OR=1.03, 95%CI:1.01 ~ 1.05) were independent predictors for poor prognosis among HTM patients (Table 3).

**Table 3:** Multivariate stepwise regression analysis of prognostic risk factors for in-hospital mortality in patient with HTM.

Variables	Univariate analysis			Multivariate stepwise regression analysis		
	β	OR (95%CI)	P	β	OR (95%CI)	P
Elevated creatinine						
no		1.000 (Reference)			1.000 (Reference)	
yes	1.708	5.520 (2.135 ~ 13.759)	0.001			
Breathlessness						
no		1.000 (Reference)			1.000 (Reference)	
yes	1.302	3.678 (1.714 ~ 7.710)	0.001	1.757	5.797 (2.455 ~ 13.689)	0.001
TG	0.403	1.497 (1.077 ~ 2.080)	0.016			
PT	0.233	1.263 (1.118 ~ 1.448)	0.001			
WBC	0.097	1.102 (1.004 ~ 1.204)	0.034			
APRI	0.031	1.032 (1.014 ~ 1.054)	0.001	0.026	1.026 (1.009 ~ 1.048)	0.009
TB	0.026	1.027 (1.012 ~ 1.043)	0.001	0.017	1.017 (1.004 ~ 1.034)	0.021
CRP	0.009	1.009 (1.004 ~ 1.015)	0.001	0.008	1.008 (1.002 ~ 1.014)	0.013
Hb	-0.021	0.979 (0.964 ~ 0.994)	0.007	-0.019	0.981 (0.963 ~ 0.998)	0.034

OR: Odds Ratio, CI: Confidence Interval; WBC, White Blood Cell count; Hb, Hemoglobin; TB, Total Bilirubin; CR, Creatinine; TG, Triglycerides; CRP, C-reactive Protein; PT, Prothrombin Time; APRI, aminotransferase/platelet ratio index.

### Construction of an individualized prediction model

**Fig 2:**
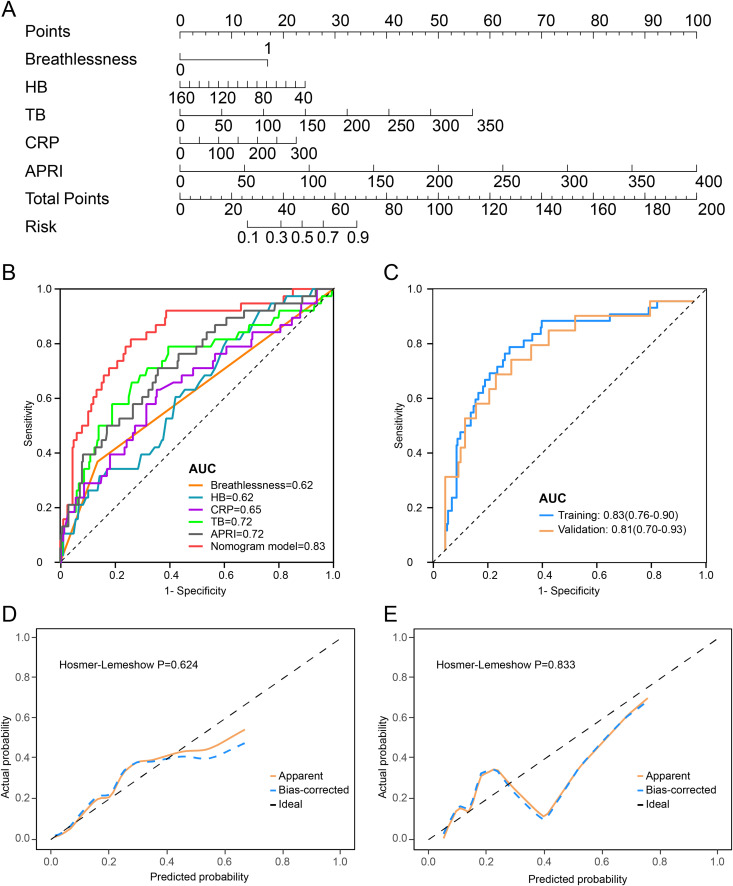
Development and validation of the Predictive Nomogram Model. A: Nomogram for predicting in-hospital mortality in HTM patients in the training set. B: ROC analysis of individual factor and combined model in predicting outcome of patients with HTM. C: ROC curves of the training set and validation set. D-E: The calibration plot of nomogram model in the training set (D) and validation set (E).

A nomogram was developed by integrating the aforementioned crucial clinical features. With breathlessness, decreased Hb, elevated total bilirubin, CRP and APRI, the odds of death were increased, as shown by the probability of adverse outcome in the model (Fig 2). Furthermore, a receiver operating characteristic (ROC) curve was performed to investigate the discrimination of the nomogram. It was observed that the area under the curve (AUC) for the nomogram in predicting death was 0.83 (95% CI: 0.76-0.90) in the training set and 0.81 (95% CI: 0.70-0.93) in the validation set, demonstrating a robust discriminatory ability of the model (S1 Table). As shown in Fig 2D-2E, the model demonstrated good calibration in both the training and validation sets, with Hosmer‒Lemeshow test p-values of 0.624 and 0.833, respectively. Additionally, the decision curve analysis (DCA) indicated that the model provided significant net benefits for patients experiencing poor outcomes (S2 Fig).

For practical implementation, we developed an Excel-based risk calculator (S1 File), allowing clinicians to input key parameters (e.g., Hb, TB, APRI) and obtain instant mortality risk scores. To assess the robustness of our nomogram against potential overfitting given the retrospective design, we performed bootstrap internal validation with 500 resamples on the training cohort. The mean area under the curve (AUC) derived from bootstrap sampling was 0.838 (95% CI: 0.762–0.906), which remained close to the apparent AUC of 0.83. Using the optimism-correction procedure, the bias-corrected AUC was 0.811, with an optimism estimate of only 0.02 (S2 Table). These results indicate that the model is not substantially overfitted and maintains stable discriminative performance.

## Discussion

TM is an opportunistic pathogenic fungus that usually causes invasive infections in those with compromised immune systems, in whom the onset of disease is insidious and mortality is high. In PWH, the mortality rate from TM infection is higher than that of most other AIDS-related diseases, ranging from 8% to 40% [6]. In this study, we examined the clinical characteristics and treatment outcomes of patients with HTM from three general hospitals in eastern China. It was found that of 431 eligible participants, 55 patients died during hospitalization, with an in-hospital mortality rate of 12.76%. Additionally, breathlessness, Hb, TB, CRP, APRI were demonstrated as reliable predictors of in-hospital mortality in patients with HTM. A nomogram to predict the in-hospital mortality risk of HTM was developed by integrating the mentioned above five factors, with an area under the curve (AUC) of 0.83 (95% CI: 0.76–0.90), sensitivity of 82%, and specificity of 74%. The nomogram model also demonstrated good discriminatory performance in the internal validation cohort, with an AUC of 0.81 (95% CI: 0.70–0.93), a sensitivity of 59%, and a specificity of 87%. By adjusting the threshold to 0.089 (optimized for the validation set), sensitivity improved to 71% with acceptable specificity (80%).

To our knowledge, this is the first study to develop and validate a model for predicting the risk of in-hospital mortality of patients with HTM. This model facilitates the dynamic prognostic stratification of HTM patients by integrating clinically accessible laboratory markers and clinical symptoms. In this study, the most significant prognostic indicator for HTM patients was the aspartate aminotransferase-to-platelet ratio index (APRI), with an area under the curve (AUC) of 0.72 (95% CI: 0.63–0.81). APRI, which consists of AST and PLT, has received widespread attention and was regarded as an effective marker for assessing liver fibrosis in patients with chronic liver disease [14,15]. Recent studies have identified the APRI as an independent prognostic risk factor in patients with various infectious diseases, including dengue, COVID-19, Klebsiella pneumoniae liver abscess, and malaria [16–19]. Indeed, APRI serves as a quantitative biomarker for hepatic function, demonstrating significant clinical utility in critical care settings. In severe systemic conditions such as multiple organ dysfunction syndrome and septic shock, secondary hepatic injury resulting from hepatocyte damage often presents with elevated AST levels thereby driving dynamic fluctuations in APRI [20]. A recent study by Shi et al [21] indicated that liver injury is common in critical cases of HTM and increased levels of AST and TB are associated with poor prognosis of HTM, which is in line with the finding of our study. In the training cohort of this study, it was observed that, compared to survivors, deceased patients had higher levels of AST and TB, longer prothrombin time, and lower levels of albumin and cholinesterase. In the final model, after adjusting for other confounding factors, higher APRI and bilirubin levels remained independently associated with increased mortality risk of HTM patients. Notably, elevated total bilirubin was identified as the second most significant factor contributing to the risk of death in HTM patients in this study. This suggests that the degree of liver injury may be closely related to the prognosis of HTM patients. In the latent infection model of *Talaromyces marneffei* constructed by Chen et al., the liver has been identified as a critical reservoir for latent infection [22]. Furthermore, mechanistic investigations have demonstrated that *Talaromyces marneffei* induces pyroptosis in hepatocytes through activation of the AIM2-caspase-1/-4-GSDMD axis, which in turn leads to liver damage [23].

In addition, it was found that the levels of Hb and PLT were significantly lower in the deceased group compared to the survival group, which is consistent with prior studies [1]. Furthermore, Hb levels were identified as an independent risk factor for HTM mortality. Studies have demonstrated that microbial infections can lead to leukopenia, anemia, and thrombocytopenia by inhibiting bone marrow function [24,25]. Wang et al. reported on 31 HTM patients who underwent bone marrow biopsy, and found that the incidences of granulocyte, erythrocyte, and megakaryocyte dysplasia were 67.7%, 58.1%, and 9.7%, respectively [26]. Thus, for HTM patients, monitoring complete blood count parameters, particularly Hb and PLT levels, is essential for identifying critical cases in a timely manner.

CRP and breathlessness were two other independent factors predicting the prognosis of HTM patients in this study. CRP, a well-known classic inflammatory marker, was closely associated with all-cause mortality in advanced HIV infected individuals [27]. Lu et al. also reported that in subjects with TM bloodstream infection, the level of CRP in the poor prognosis group was significantly higher than those in the survival group [11]. In our study, elevated CRP was associated with an increased risk of patient mortality, further supporting its role as a predictive factor for HTM prognosis. Breathlessness, a self-reported symptom in this study, is a fundamental homeostatic warning signal of inadequate alveolar ventilation. In PWH, *Talaromyces marneffei* frequently causes systemic disseminated infections and patients with involvement of the lower respiratory tract may present with symptoms such as fever, cough, dyspnea and respiratory failure [2,28]. Additionally, fungal dissemination can trigger a systemic inflammatory response, leading to metabolic acidosis, which may exacerbate breathlessness. A study involving 127 HTM patients found that breathlessness was an independent predictor of mortality. Moreover, another study reported that breathlessness and a lower platelet count predicted poor in-hospital outcomes [1], which is in agreement with our findings. However, self-reported breathlessness may be subject to cultural or cognitive bias, and future studies should incorporate objective respiratory rate monitoring (>24 breaths per minute) to enhance reporting reliability.

It is notable that there is an inconsistency between our current findings and previous studies regarding the predictive value of CD4 + T cells, CD8 + T cells, the G test, and albumin [11,21]. First, differences in study populations could contribute to the inconsistencies. In our cohort, the median CD4 + T cell count was only 13 (IQR: 4.00, 38.01), which may explain why differences in immune cell counts between survivors and non-survivors were not significant. Additionally, sample size and statistical power may also play a role. Lastly, the potential confounding effects of other variables in the multivariate analysis should be considered, as they may have masked the predictive value of albumin in our study.

Our study has several limitations. The retrospective design may introduce inherent bias, although internal validation suggested minimal overfitting and stable discrimination. Several potential factors, such as BMI, procalcitonin, underlying diseases, quantitative fungal burden, and HIV viral load, were not included in our analysis; incorporating them might further improve predictive accuracy. Additionally, this multicenter study was limited to East China, and the lack of external validation restricts generalizability to other regions. Therefore, our nomogram should be considered exploratory and not used clinically without independent validation. We encourage other groups to validate it using their own cohorts, and we have provided the full prediction formula and an Excel calculator to facilitate such efforts. Furthermore, because the study spanned a decade during which antifungal and supportive care evolved, the model’s predictive accuracy may differ in future cohorts; we therefore recommend periodic re‑validation when applying the nomogram to newly diagnosed patients. Finally, while the model’s AUC is acceptable, its sensitivity at the training‑derived threshold is modest. However, the threshold can be adjusted according to clinical priorities (e.g., a lower threshold for screening), and decision curve analysis supports net benefit across a wide range of thresholds.

## Conclusion

In conclusion, this is the first study to develop and validate a nomogram for predicting in-hospital mortality in patients with HTM based on multicenter data. The nomogram integrates factors such as symptom of breathlessness, Hb, TB, APRI, and CRP, and has been internally validated. In addition, the model demonstrates excellent discriminatory power, calibration, and clinical validity, allowing for the identification of patients at higher risk of in-hospital mortality. As a useful tool for risk assessment, the developed predictive models will be of great value in the identification, stratification and management of HTM patients.

## Supporting information

S1 Fig Variables selection using the LASSO logistic regression. LASSO, least absolute shrinkage and selection operator.
(TIF)

S2 Fig Decision Curve Analysis of the Predictive Model in the Training (A) and Validation sets (B). The green line represents the assumption of no HTM patient dies during hospitalisation, while the red line assumes that all patients die during hospitalization. The blue line corresponds to the risk nomogram. The analysis was conducted on both the training set (A) and the validation set (B).
(TIF)

S1 Table Performance of the Predictive Model in the Training and Validation Cohorts. APRI, aminotransferase/platelet ratio index; AUC, Area Under the Curve; CI, Confidence Interval; CRP, C-reactive protein; Hb, Hemoglobin; PPV, Positive Predictive Value; NPV, Negative Predictive Value, TB, Total bilirubin.
(DOCX)

S2 Table Bootstrap internal validation of the nomogram. AUC, Area Under the Curve; CI, Confidence Interval.
(DOCX)

S1 File Risk Calculator for HTM Patients. (XLSX)

S2 File The original data for statistic in our study. (XLSX)
