# Supplementary figures and images for: Multivariate predictive model for predicting in-hospital mortality in HIV-associated talaromycosis: a multicenter retrospective study
Source: PLoS Negl Trop Dis. 2026 Jun 8;20(6):e0014432. doi: 10.1371/journal.pntd.0014432 (PMC13262935; doi:10.1371/journal.pntd.0014432)

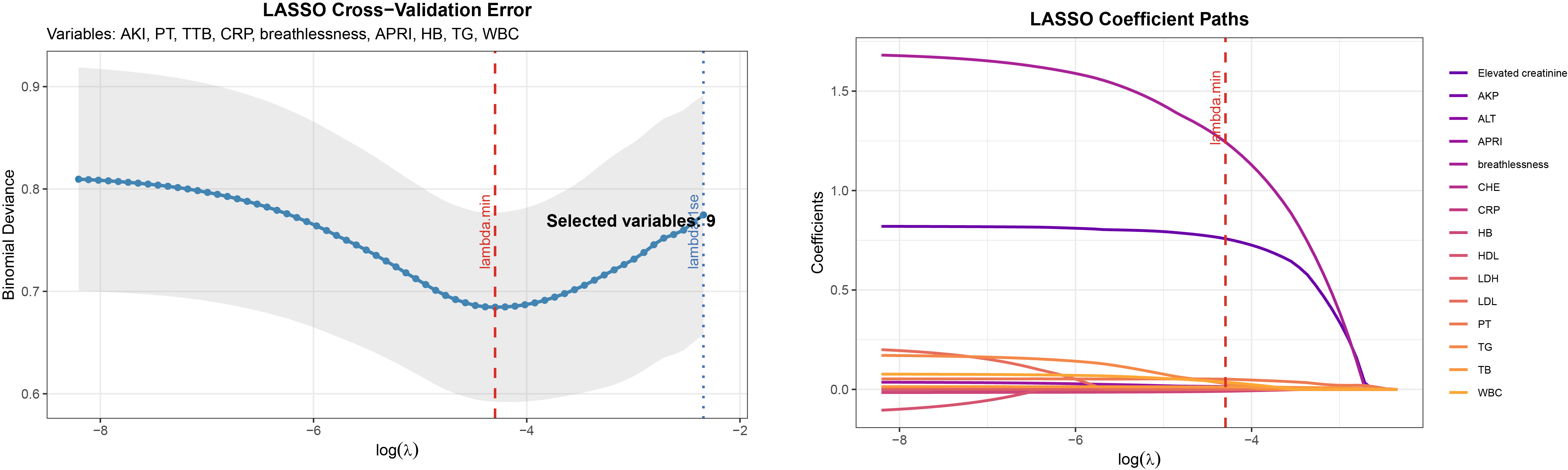

Supplement: S1 Fig — LASSO, least absolute shrinkage and selection operator. (TIF) [file pntd.0014432.s001.tif]

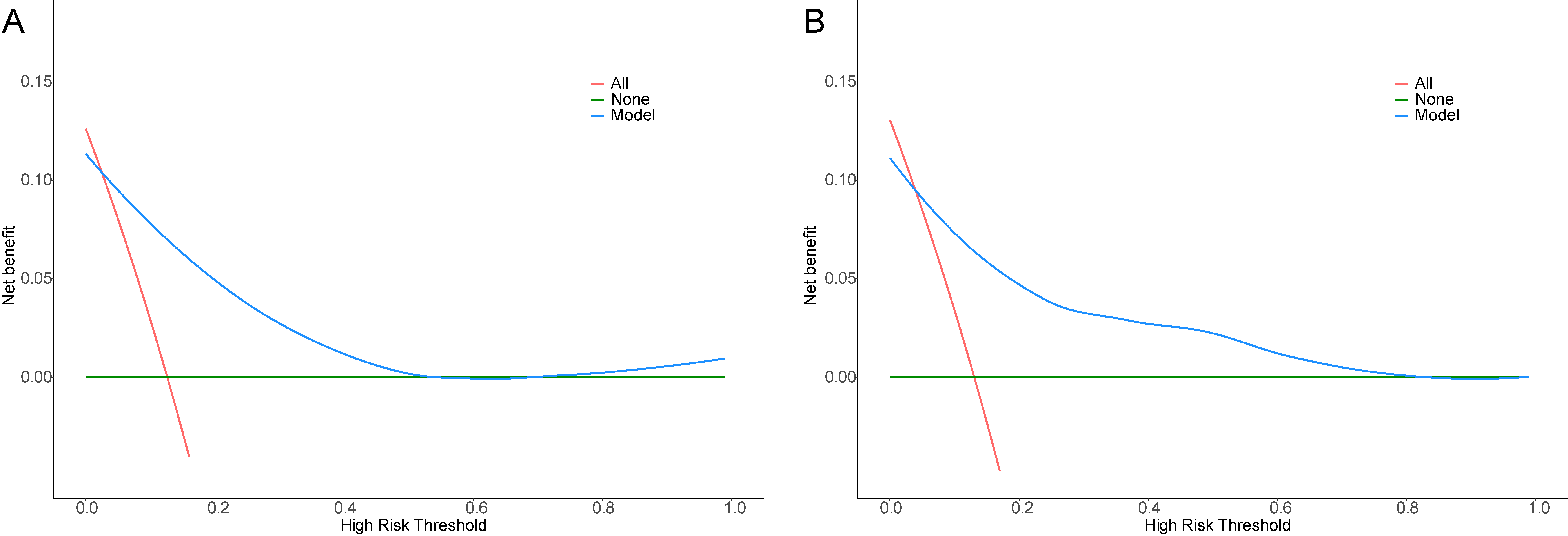

Supplement: S2 Fig — The green line represents the assumption of no HTM patient dies during hospitalisation, while the red line assumes that all patients die during hospitalization. The blue line corresponds to the risk nomogram. The analysis was conducted on both the training set (A) and the validation set (B). (TIF) [file pntd.0014432.s002.tif]
