# Supplementary material for: Multivariate predictive model for predicting in-hospital mortality in HIV-associated talaromycosis: a multicenter retrospective study
Source: PLoS Negl Trop Dis. 2026 Jun 8;20(6):e0014432. doi: 10.1371/journal.pntd.0014432 (PMC13262935; doi:10.1371/journal.pntd.0014432)
Supplement: S1 Table — APRI, aminotransferase/platelet ratio index; AUC, Area Under the Curve; CI, Confidence Interval; CRP, C-reactive protein; Hb, Hemoglobin; PPV, Positive Predictive Value; NPV, Negative Predictive Value, TB, Total bilirubin. (DOCX) [file pntd.0014432.s003.docx]

# S1 Table. Performance of the Predictive Model in the Training and Validation Cohorts

| Data | AUC (95%CI) | Accuracy (95%CI) | Sensitivity (95%CI) | Specificity (95%CI) | PPV (95%CI) | NPV (95%CI) | cut off |
| --- | --- | --- | --- | --- | --- | --- | --- |
|  |  |  |  |  |  |  |  |
| Train | 0.83 (0.76-0.90) | 0.75 (0.70-0.80) | 0.82 (0.69 - 0.94) | 0.74 (0.69 - 0.79) | 0.31 (0.22 - 0.40) | 0.97 (0.94 - 0.99) | 0.108 |
| Test | 0.81 (0.70-0.93) | 0.83 (0.76-0.89) | 0.59 (0.35 - 0.82) | 0.87 (0.80 - 0.93) | 0.40 (0.21 - 0.59) | 0.93 (0.89 - 0.98) | 0.108 |

APRI, aminotransferase/platelet ratio index; AUC, Area Under the Curve; CI, Confidence Interval; CRP, C-reactive protein; HB, Hemoglobin; PPV, Positive Predictive Value; NPV, Negative Predictive Value, TB, Total bilirubin
