# Supplementary material for: Multivariate predictive model for predicting in-hospital mortality in HIV-associated talaromycosis: a multicenter retrospective study
Source: PLoS Negl Trop Dis. 2026 Jun 8;20(6):e0014432. doi: 10.1371/journal.pntd.0014432 (PMC13262935; doi:10.1371/journal.pntd.0014432)
Supplement: S2 Table — AUC, Area Under the Curve; CI, Confidence Interval. (DOCX) [file pntd.0014432.s004.docx]

**S2 Table. Bootstrap internal validation of the nomogram**

| **Method** | **AUC** | **95% CI** | **Optimism estimate** |
| --- | --- | --- | --- |
| Apparent AUC (original training set) | 0.83 | 0.76–0.90 | – |
| Bootstrap mean AUC (500 resamples) | 0.84 | 0.76–0.91 | – |
| Optimism-corrected AUC | 0.81 | 0.73-0.89 | 0.02 |

AUC, Area Under the Curve; CI, Confidence Interval;
